# Evaluation of PD-L1 and B7-H3 expression as a predictor of response to adjuvant chemotherapy in bladder cancer

**DOI:** 10.1186/s12894-022-01044-1

**Published:** 2022-06-24

**Authors:** Ahmed M. Mahmoud, Igor Frank, Jacob J. Orme, Roxane R. Lavoie, Prabin Thapa, Brian A. Costello, John C. Cheville, Sounak Gupta, Haidong Dong, Fabrice Lucien

**Affiliations:** 1 Department of Urology, Mayo Clinic, Guggenheim 4-97, 200 1st Street SW, Rochester, MN 55905 USA; 2 Department of Medical Oncology, Mayo Clinic, Rochester, MN USA; 3 Department of Pathology and Laboratory Medicine, Mayo Clinic, Rochester, MN USA

**Keywords:** Bladder cancer, Adjuvant chemotherapy, Immunohistochemistry, PD-L1, B7-H3

## Abstract

**Introduction and objectives:**

PD-L1 and B7-H3 have been found to be overexpressed in urothelial carcinoma (UC) of the urinary bladder. Recent studies have also demonstrated that B7-H3 and PD-L1 can promote resistance to platinum-based drugs but the predictive value of B7-H3 expression in patients treated with platinum-based chemotherapy is unknown. This study aims to investigate the association of PD-L1 and B7-H3 tumor expression with oncological outcomes in patients who underwent radical cystectomy (RC) and received subsequent adjuvant chemotherapy.

**Materials and methods:**

Immunohistochemistry was performed on paraffin-embedded sections from bladder and lymph node specimens of 81 patients who had RC for bladder cancer. PD-L1 and B7-H3 expression on tumor cells was assessed by immunohistochemistry in both primary tumors and lymph node specimens. Association with clinicopathologic outcomes was determined using Fisher's exact test and postoperative survival using Kaplan–Meier survival curves and Cox regression model.

**Results:**

B7-H3 expression in cystectomy specimens was more common than PD-L1 expression (72.8% vs. 35.8%). For both markers, no association was found with pathologic tumor stage, lymph node (LN) status, and histological subtype. Similar findings were observed for double-positive tumors (PD-L1^+^B7-H3^+^). Concordance between the primary tumor and patient-matched lymph nodes was found in 76.2% and 54.1% of patients for PD-L1 and B7-H3, respectively. PD-L1 tumor expression was not associated with oncologic outcomes. However, B7-H3 expression was associated with recurrence-free survival (HR: 2.38, 95% CI 1.06–5.31, *p* = 0.035) and cancer-specific survival (HR: 2.67, 95% CI 1.18–6.04, *p* = 0.019).

**Conclusions:**

In our single institutional study, B7-H3 is highly expressed in patients with UC treated with adjuvant chemotherapy and it was associated with decreased recurrence-free survival and cancer-specific survival. Pending further validation in larger cohorts, B7-H3 expression may function as a predictor of response to adjuvant chemotherapy and thus be useful in patient and regimen selection.

**Supplementary Information:**

The online version contains supplementary material available at 10.1186/s12894-022-01044-1.

## Introduction

Urothelial carcinoma of the bladder (UCB) is the second most prevalent genitourinary cancer, with an approximately 83,730 new cases and 17,200 UCB-related mortalities in the United States in 2021 [1]. Radical cystectomy is the primary therapeutic option for patients diagnosed with muscle-invasive bladder cancer. For patients presenting high-risk features (≥ pT3 or N+) and eligible for systemic therapy, adjuvant chemotherapy or immunotherapy may provide overall and disease-free survival benefit [2, 3].

While platinum-based drugs are used for the treatment of various cancers, intrinsic and acquired resistance is a major cause of disease recurrence. Extensive research has been conducted to identify molecular determinants of platinum resistance [4]. Immune checkpoint molecules PD-L1 and B7-H3 are well recognized for their role in tumor immune evasion but recent work has underlined a potential role in resistance to platinum-based drugs. In response to drug treatment, PD-L1 promotes DNA damage repair and reduces the efficacy of cisplatin in preclinical mouse models [5, 6]. B7-H3 knockout was also associated with increased sensitivity to cisplatin in vitro but further studies are warranted to validate these findings using in vivo tumor models [7, 8]. This prior work supports the need to determine whether PD-L1 and B7-H3 can serve as predictive markers of response to platinum-based therapies in urothelial carcinoma.

Tumor PD-L1 expression and its association with oncological outcomes have been extensively investigated in urothelial carcinoma. PD-L1 expression has been linked to a higher tumor grade and a lower survival rate in urothelial malignancies [9]. It has previously been established as an independent predictor of local progression in response to Bacillus Calmette–Guerin (BCG) [10]. The role of PD-L1 as a potential biomarker in predicting oncologic outcomes in bladder cancer after radical cystectomy (RC) is still debated, with contradictory evidence [9, 11, 12]. One study with a limited sample size (N = 42) found no association between PD-L1 status and response to adjuvant chemotherapy [13]. B7-H3 is overexpressed in bladder cancer irrespective of tumor stage, but it has not been shown to predict recurrence and mortality post-cystectomy [9, 14]. To our knowledge, the predictive value of B7-H3 in bladder cancer patients treated with adjuvant chemotherapy has not been established yet.

In the present study, PD-L1 and B7-H3 expression in RC specimens of 81 high-risk bladder cancer patients who received adjuvant chemotherapy was evaluated. The primary analysis included examination of PD-L1 and B7-H3 expression in primary tumors and patient-matched lymph node tissue. In a secondary analysis, we examined the association of PD-L1 and B7-H3 expression with clinical outcomes.

## Materials and methods

### Patient cohort

With the approval of the Mayo Clinic Institutional Review Board (IRB# 19-003470), we identified 81 patients who underwent radical cystectomy followed by adjuvant platinum-based chemotherapy between 1985 and 2019. Our database was used to collect baseline clinicopathological variables such as age, gender, metastasis and recurrence status, tumor stage, histological type, lymph node (LN) status, and clinical follow-up data. We excluded patients who received preoperative (neoadjuvant) chemotherapy, immunotherapy, or radiation and included only patients who received adjuvant chemotherapy including cisplatin, carboplatin, CMV, and MVAC. Patient characteristics are listed in Table 1. A subset of patients with available metastatic lymph node specimens (PD-L1 = 43 patients, B7-H3 = 59 patients) was included to compare PD-L1 and B7-H3 expression in primary tumors and patient-matched metastasis. All experiments were carried out in accordance with relevant guidelines and regulations.

**Table 1:** Patient characteristics

Feature	n (%)
Age, mean ± SD, years	61.4 ± 9.84
Gender	
Male	64 (79%)
Female	17 (21%)
Tumor staging	
pTis/Cis	1 (1.2%)
pT1	4 (4.9%)
pT2a	5 (6.2%)
pT2b	14 (17.3%)
pT3a	33 (40.8%)
pT3b	19 (23.5%)
pT4a	4 (4.9%)
pT4b	1 (1.2%)
LN status	
Nx	1 (1.2%)
N0	12 (14.8%)
N1	22 (27.2%)
N2	38 (46.9%)
N3	8 (9.9%)
Metastasis	
Yes	36 (44.4%)
No	45 (55.6%)
Recurrence	
Yes	39 (48.1%)
No	42 (51.9%)
Death	
Yes	52 (64.2%)
No	29 (35.8%)
Histological type	
Pure Urothelial carcinoma	69 (85.2%)
Micropapillary carcinoma	4 (4.9%)
Sarcomatoid carcinoma	6 (7.5%)
Adenocarcinoma	1 (1.2%)
No residual invasive tumor	1 (1.2%)
Chemotherapy type	
Cisplatin	36 (44.4%)
MVAC	18 (22.2%)
CMV	17 (20.9%)
Carboplatin	10 (12.3%)

*Abbreviation: Cis=* Carcinoma in situ

### Immunohistochemistry and scoring

Tissue slides were reviewed by an independent expert urologic pathologist (JC) to identify tumor-positive sections amenable for immunohistochemistry. Tissue sectioning (5 microns) was performed at the Pathology Research Core (Mayo Clinic, Rochester, MN). B7-H3 staining was conducted in our laboratory (D9M2L clone, Cell Signaling Technology, #14058S) and antibody specificity was validated using B7-H3 expressing and B7H3-knockout RH30 (rhabdomyosarcoma) tumor xenografts (Additional file 1: Fig. S1A). PD-L1 staining was performed by the Mayo Clinic Pathology Research Core (PRC) using the PD-L1 antibody E1L3N clone (Cell Signaling Technology, #13684S). Negative and positive controls for PD-L1 staining are presented in Additional file 1: Fig. S1B. Detailed IHC staining protocols for both PD-L1 and B7-H3 staining are presented in Additional file 1.

**Fig. 1:**
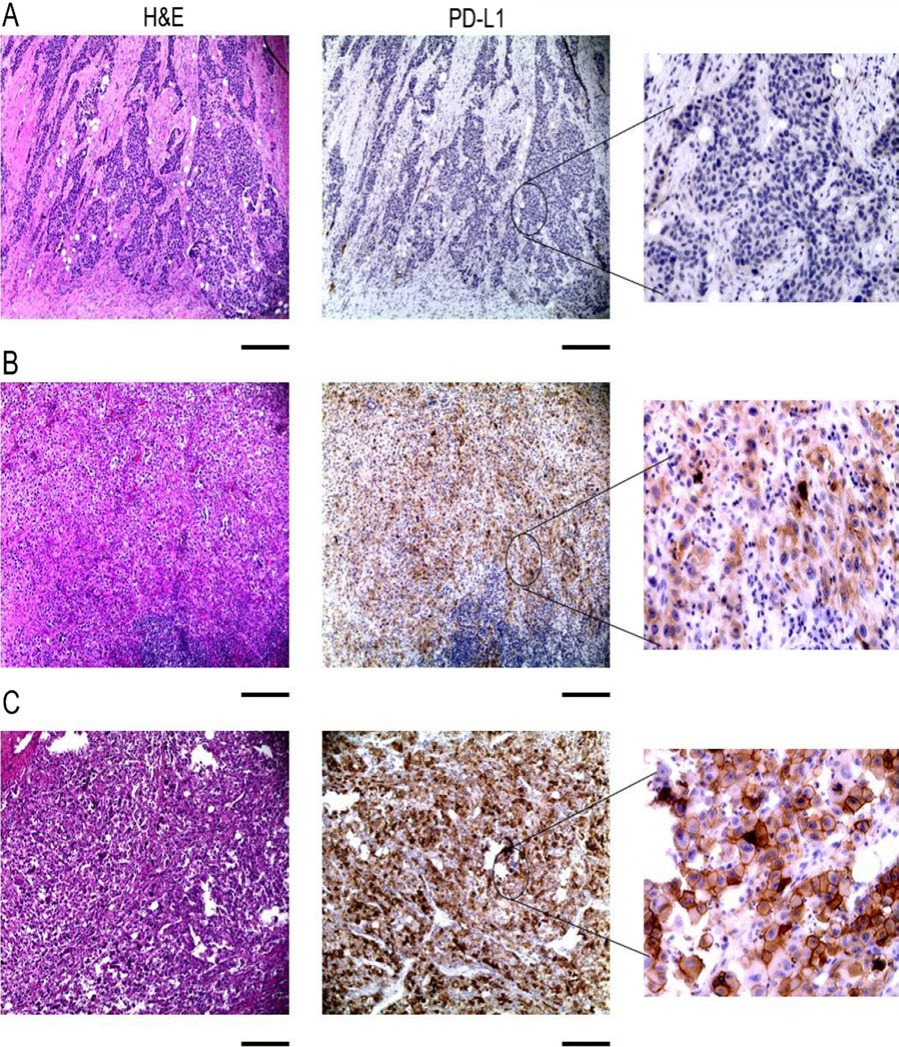
Representative images of PD-L1 staining in radical cystectomy specimens. **A** Negative expression of PD-L1, **B** Low expression of PD-L1, **C** High expression of PD-L1. Size bar = 500 µm

**Fig. 2:**
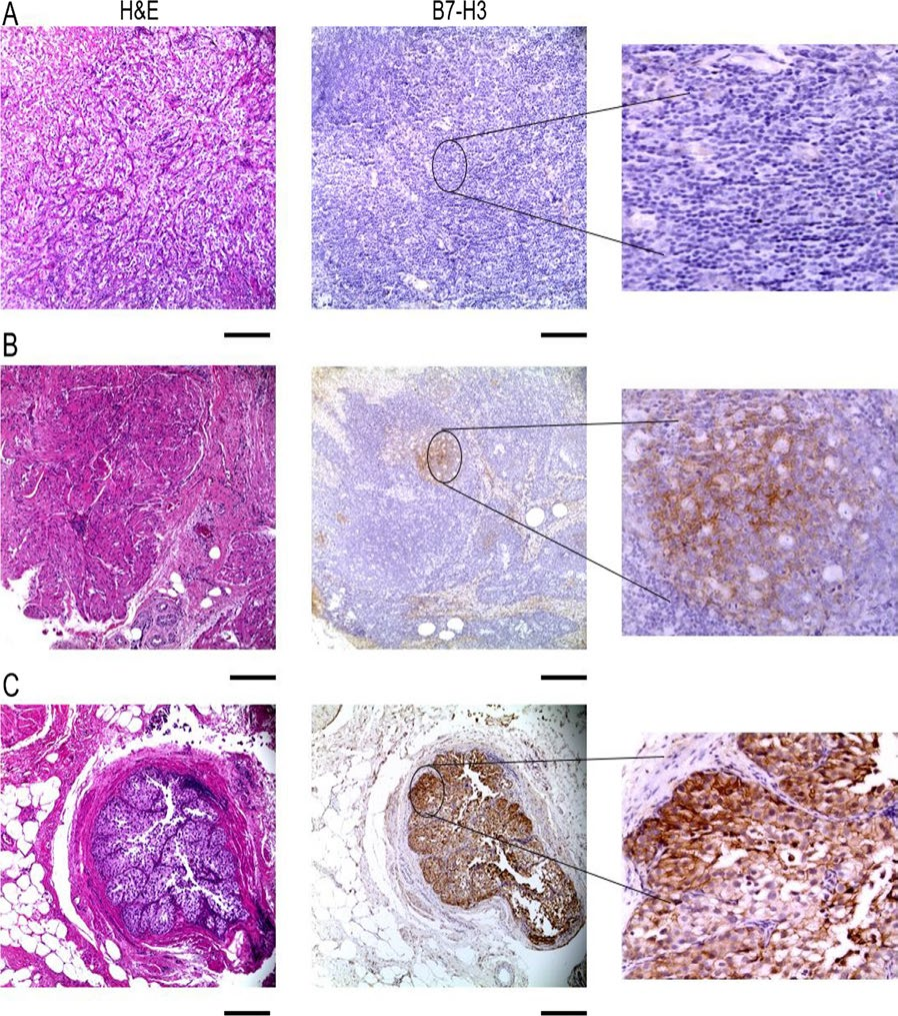
Representative images of B7-H3 staining in radical cystectomy specimens. **A** Negative expression of B7-H3, **B** Low expression of B7-H3, **C** High expression of B7-H3. Size bar = 500 µm

Tumors were assessed by two independent expert urologic pathologists (SG and JC) blinded to clinical data. A standardized H-score was utilized, ranging from 0 (no cell membrane expression) to 300 (100% cells positive for strong membrane expression). The H-score used the following formula: (1 × (% cells weak expression) + 2 × (% cells with moderate expression) + 3 × (% cells strong expression). The H-score evaluated expression only in tumor cells. Degree of concordance of H-scoring between both pathologists is presented in Additional file 1: Fig. S2. The average of the H-scores obtained from both pathologists was used for statistical analyses and was grouped as follows: 0: negative (H-score = 0), 1: Low (H-score ≥ 1 and < 120), 2 = high (H-score ≥ 120) (Figs. 1 and 2).

### Statistical analysis

Fisher's exact test was used to assess the associations of PD-L1 and B7-H3 with clinicopathological characteristics. Depending on the outcome measure of interest, the period of patient follow-up was estimated from the date of cystectomy to the date of recurrence, death, or last known follow-up. The relationship between PD-L1 and B7-H3 expression and clinical outcomes (recurrence-free survival, cancer-specific survival and overall survival) was evaluated using univariate Cox proportional hazards regression models and presented using hazard ratios (HR) and 95% confidence intervals (CI). The Kaplan–Meier method was used to calculate recurrence-free, cancer-specific, and overall survival probabilities following cystectomy, and differences in survival were assessed using the log-rank test. All tests were two-sided, and a *p* value of < 0.05 was considered statistically significant. Statistical analyses were done using the SPSS v.26 (SPSS Inc., IBM Corp., Armonk, NY).

## Results

### PD-L1 and B7-H3 expression in radical cystectomy and patient-matched lymph nodes

Between 1985 and 2019, we identified 127 patients who underwent radical cystectomy and received adjuvant chemotherapy, of whom 81 (63.8%) had paraffin-embedded tissue blocks accessible for examination. Table 1 describes patient and tumor characteristics. PD-L1 expression was observed in 29/81 (35.8%) of RC specimens (Table 2). The degree of expression was found to be low [1] and high [2], in 21 (25.9%) and 8 (9.9%) samples, respectively (Fig. 1). In tumor-positive lymph nodes, PD-L1 expression was negative in 29/42 (69.1%) and positive in 13/42 (30.9%). B7-H3 expression was observed in 59/81 (72.8%) of RC specimens with low expression in 42/81 (51.9%) and high expression in 17/81 (20.9%) (Fig. 2) (Table 2). B7-H3 positivity was observed in 35/59 (59.3%) of LN specimens. The degree of concordance of PD-L1 expression in RC and patient-matched LN was 76.2% (Table 3). Compared to PD-L1, B7-H3 expression was not highly concordant between primary tumors and lymph nodes with only 54.1% being concordant. In radical cystectomy specimens, we found that only 30.9% and 14.8% were positive and negative for both PD-L1 and B7-H3, respectively. Indeed, a high number of specimens were B7-H3 positive and PD-L1 negative (41.9%).

**Table 2:** PD-L1 and B7-H3 RC and LN distribution

Feature	n (%)	Feature	n (%)
*PD-L1 RC H-score*		*B7-H3 RC H-score*	
Negative	52 (64.2%)	Negative	22 (27.2%)
Low 1–120	21 (25.9%)	Low 1–120	42 (51.9%)
High ≥ 120	8 (9.9%)	High ≥ 120	17 (20.9%)
*PD-L1 LN H-score*		*B7-H3 LN H-score*	
Negative	29 (69.1%)	Negative	24 (40.7%)
Low 1–120	9 (21.4%)	Low 1–120	33 (55.9%)
High ≥ 120	4 (9.5%)	High ≥ 120	2 (3.4%)

0: No expression, 1: weak/medium expression, 2: High expression

*RC* Radical cystectomy, *LN* lymph node

**Table 3:** Concordance of tumor (RC) and lymph node (LN) expression of PD-L1 and B7-H3

		PD-L1 RC				B7-H3 RC				PD-L1 RC	
		Positive	Negative			Positive	Negative			Positive	Negative
PD-L1 LN	Positive	11 (26.2%)	4 (9.5%)	B7-H3 LN	Positive	25 (42.4%)	10 (16.9%)	B7-H3 RC	Positive	25 (30.9%)	34 (41.9%)
	Negative	6 (14.3%)	21 (50%)		Negative	17 (28.8%)	7 (11.9%)		Negative	10 (12.4%)	12 (14.8%)

### Association of PD-L1 and B7-H3 expression with clinicopathological features

Association of PD-L1 and B7-H3 with clinicopathological characteristics is summarized in Table 4. There was no association between PD-L1 and B7-H3 expression and clinicopathological features.

**Table 4:** Association of tumor PD-L1 and B7-H3 expression with clinicopathological features

Feature	Tumor PD-L1 expression		P*	Tumor B7-H3 expression		P*
	High (n = 8)	Low/negative (n = 73)		High (n = 17)	Low/negative (n = 64)	
Age			0.932			0.818
Mean (SD)	61 (7.1)	61.3 (9.9)		61.4 (7.7)	60.8 (10.5)	
Median	61.9	61.6		60.6	62.7	
Gender			0.677			0.345
Male	7 (87.5%)	56 (76.7%)		15 (88.2%)	49 (76.6%)	
Female	1 (12.5%)	17 (23.3%)		2 (11.8%)	15 (23.4%)	
Tumor stage			0.424			0.242
≤ T2b	1 (87.5%)	24 (32.9%)		3 (17.6%)	22 (34.4%)	
≥ T3a	7 (12.5%)	49 (67.1%)		14 (82.4%)	42 (65.6%)	
LN status			0.084			0.351
Positive	5 (62.5%)	63 (86.3%)		13 (76.5%)	55 (85.9%)	
Negative	3 (37.5%)	10 (13.7%)		4 (23.5%)	9 (14.1%)	
Histological type			0.352			0.054
Pure UC	8 (100%)	61 (83.7%)		17 (100%)	52 (81.3%)	
Other subtypes	0 (0%)	12 (16.4%)		0 (0%)	12 (18.7%)	

*LN* Lymph node, *UC* urothelial carcinoma

^*^P values by Fisher’s exact test: *p < 0.05, **p < 0.01, ***p < 0.001

### Association of PD-L1 and B7-H3 expression with clinical outcomes

**Fig. 3:**
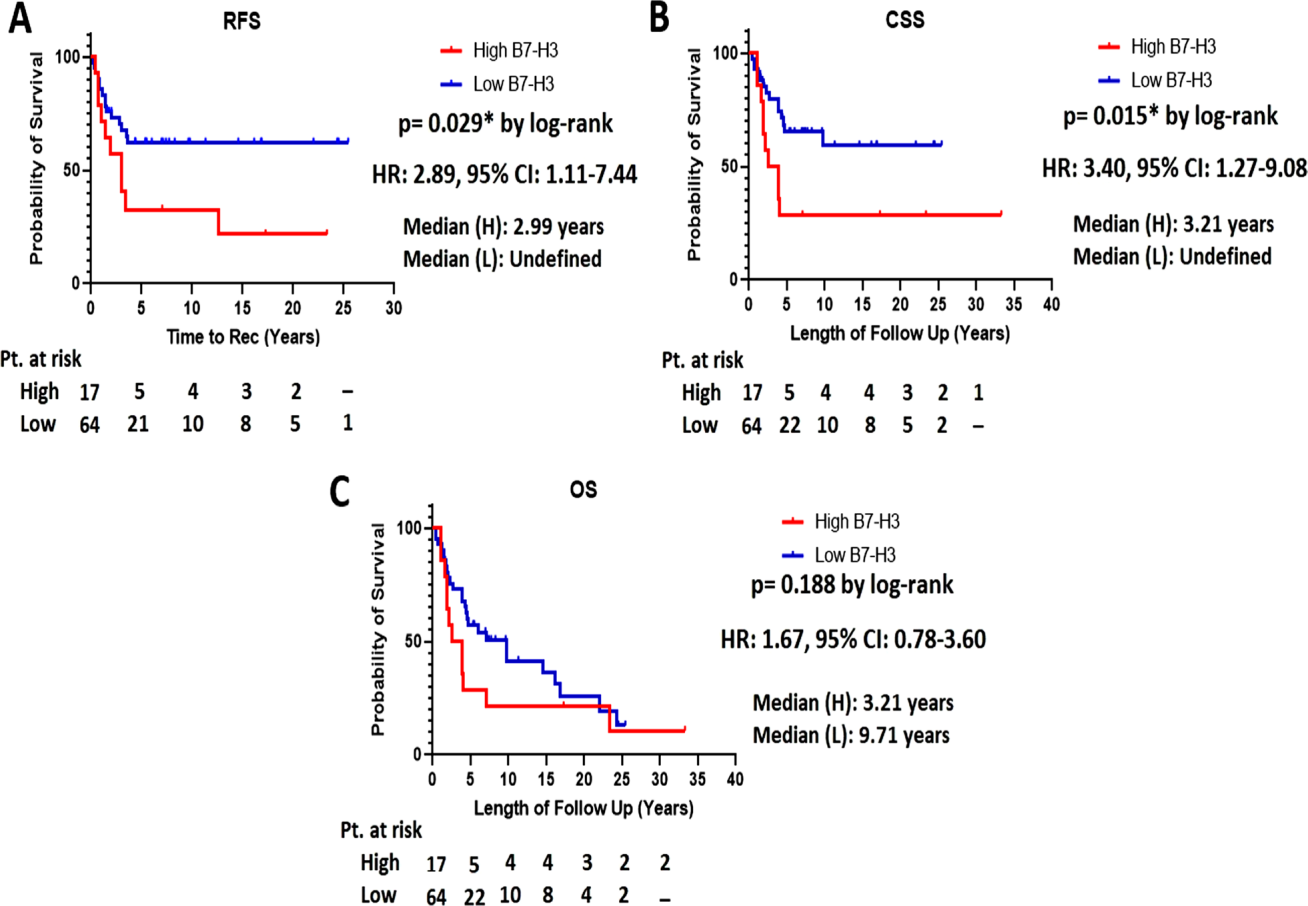
Association of B7-H3 expression with recurrence-free survival, cancer-specific survival, and overall survival. **A** Recurrence-free survival (RFS) in tumors with high and low/negative expression of B7-H3. **B** Cancer-specific survival (CSS) in tumors with high and low/negative expression of B7-H3. **C** Overall survival (OS) in tumors with high and low/negative expression of B7-H3

In the entire cohort, the median follow-up time following RC was 4.4 years (IQR, 1.97–10.58). During this time, 39 (48.1%) patients had disease recurrence, 32 (39.5%) died of cancer, and 20 (24.7%) died from other causes (Table 1). No association was found between PD-L1 expression and progression-free survival, cancer-specific survival, or overall survival (Table 5). However, B7-H3 expression was associated with lower recurrence-free survival (HR: 2.38, 95% CI 1.06–5.31, *p* = 0.035) and cancer-specific survival (HR: 2.67, 95% CI 1.18–6.04, *p* = 0.019). Kaplan–Meier survival curves for patients treated with adjuvant chemotherapy and stratified by tumor B7-H3 expression are shown in Fig. 3. At 5 years of follow-up, cancer-specific survival was 29.4% and 64.4% in B7-H3 high and low/negative tumors, respectively (*p* = 0.015) (Fig. 3A). Medial overall survival was 3.21 years for patients with B7-H3-high tumors and 9.71 years for patients with B7-H3 low tumors, but it did not reach statistical significance (*p* = 0.188) (Fig. 3B).

**Table 5:** Univariate analysis of association of PD-L1 and B7-H3 expression with clinical outcomes

Feature	RFS		CSS		OS	
	HR (95%CI)	*p* values	HR (95%CI)	*p* values	HR (95%CI)	*p* values
PD-L1 H-score ≥ 120	1.30 (0.68–2.47)	0.439	1.16 (0.57–2.35)	0.689	1.46 (0.82–2.59)	0.197
B7-H3 H-score ≥ 120	2.38 (1.06–5.31)	0.035*	2.67 (1.18–6.04)	0.019*	1.86 (0.79–3.16)	0.192

*RFS* recurrence-free survival, *DSS* disease-specific survival, *OS* Overall survival

## Discussion

To our knowledge, this study is the first to show that B7-H3 is an independent predictor of cancer mortality after cystectomy in bladder cancer patients treated with adjuvant chemotherapy. We found an association between B7-H3 expression and oncological outcomes. At the same time, we confirmed that B7-H3 is more frequently expressed in urothelial carcinoma compared to its homolog PD-L1. High PD-L1 tumor expression has been previously associated with poor outcome post-cystectomy but its predictive value in response to adjuvant chemotherapy remains debatable. Herein, we did not find any evidence of an association between PD-L1 expression and oncological outcomes. Finally, we found limited concordance in B7-H3 expression between cystectomy specimens and patient-matched lymph nodes.

Following the path of its well-known homolog PD-L1 (B7-H1), B7-H3 is actively investigated as a tumor target for anticancer therapies [15]. Highly expressed in several cancers, B7-H3 promotes tumor progression by inhibiting antitumor immune response [16, 17]. We found a high proportion of cystectomy specimens positive for B7-H3 (72.8%). This is in line with a prior study reporting 70.7% of cases with detectable B7-H3 expression (14). A second study reported only 58.6% of cases positive for B7-H3 [9]. The discrepancy in detection rates between studies can be partly explained by the use of different commercial antibodies for immunohistochemistry. Our study is unique in that we used a monoclonal antibody against B7-H3 (D9M2L clone) while previous studies were performed with a polyclonal antibody. In our cohort of bladder cancer patients with high-risk features, B7-H3 expression was consistently increased in all bladder tumor stages from organ-confined (< T3) to locally advanced (≥ T3) and metastatic disease (N+). In contrast, B7-H3 expression is very limited in normal adjacent urothelium which suggests that B7-H3 upregulation may occur at an early stage during malignant transformation [14]. Regulation of B7-H3 expression has not been well elucidated but few studies have provided valuable molecular insights into the epigenetic and oncogenic regulation of B7-H3. In glioma, hypomethylation of the B7-H3 gene promoter resulted in ectopic expression of B7-H3 [18]. Several binding sites for the oncogenic drivers c-Myc and Androgen Receptor (AR) have been found in the B7-H3 promoter [19, 20]. While binding of c-Myc was associated with transcriptional upregulation of B7-H3, binding of AR on B7-H3 promoter repressed its expression [20]. Altogether, these original findings underline the positive and negative oncogenic regulation of B7-H3 in cancer, and further studies are warranted to delineate the molecular mechanisms regulating B7-H3 expression in bladder cancer.

Besides its inherent immune function, B7-H3 harbors tumor-protecting intrinsic functions by promoting metastasis formation, angiogenesis, and chemoresistance [21–23]. Interestingly, high B7-H3 expression has been associated with resistance to platinum-based drugs oxaliplatin, gemcitabine, and cisplatin in vitro but the underlying mechanisms remain to be elucidated [7, 8, 21, 24]. Herein, we found that elevated expression of B7-H3 in primary bladder tumors was significantly associated with decreased recurrence-free survival (*p* = 0.029) and cancer-specific survival (*p* = 0.015). These findings can serve as a justification to further investigate the intrinsic functions of B7-H3 in resistance to platinum-based drugs. While PD-L1 has been previously reported to predict postoperative mortality in patients with organ-confined tumors, we did not find any association with outcome in patients treated with adjuvant chemotherapy [9, 14]. External validation is needed but our original data suggest that B7-H3 expression status may help select patients who will benefit the most from adjuvant chemotherapy. Our findings also point towards a potential role of B7-H3 as a predictive biomarker in the neoadjuvant setting for at least two reasons. While side-to-side comparison with chemotherapy is awaited, it is anticipated that adjuvant immunotherapy (nivolumab) will become the preferred treatment for patients with high-risk bladder cancer [25]. Secondly, the combination of radical cystectomy and perioperative chemotherapy is associated with significant treatment-related complications. Patients are usually more "fit" to receive platinum-based chemotherapy preoperatively, hence, B7-H3 may find great utility for patient selection in this area.

We recognize our study has a few limitations. First, this is a retrospective study with a small sample size and a possibility of selection bias. Second, it is a single institution study; thus needs further external validation. Third, immunohistochemistry used commercially available antibodies with varying sensitivities and in which the definition of optimal cut-off values for positivity can differ significantly between suppliers. Finally, four different adjuvant treatments were used for the selected patient cohort. Head-to-head comparison of different adjuvant regimens have not been reported, therefore we cannot exclude potential treatment bias that occurs in survival analyses.

## Conclusions

In our study we found that B7-H3 is highly expressed in patients with UC treated with adjuvant chemotherapy following RC and is associated with decreased recurrence-free and cancer-specific survival. Pending further validation in larger external cohorts, B7-H3 expression may function as a predictor of response to adjuvant chemotherapy and thus be useful in patient and regimen selection.

## Supplementary Information

**Additional file 1.** Supplementray figures and materials.

## Data Availability

Data are available upon reasonable request. Direct inquiries should be made to Fabrice Lucien, PhD, Mayo Clinic, Rochester (lucien-matteoni.fabrice@mayo.edu).
