# Supplementary material for: Evaluation of PD-L1 and B7-H3 expression as a predictor of response to adjuvant chemotherapy in bladder cancer
Source: BMC Urol. 2022 Jun 24;22:90. doi: 10.1186/s12894-022-01044-1 (PMC9233321; doi:10.1186/s12894-022-01044-1)
Supplement: Supplementary file 1 — Additional file 1. Supplementray figures and materials. [file 12894_2022_1044_MOESM1_ESM.docx]

**Additional file Figures**


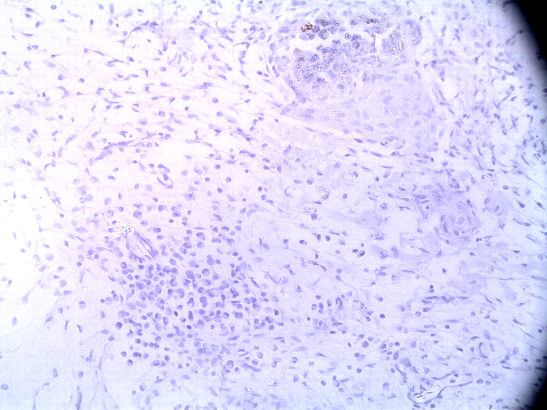

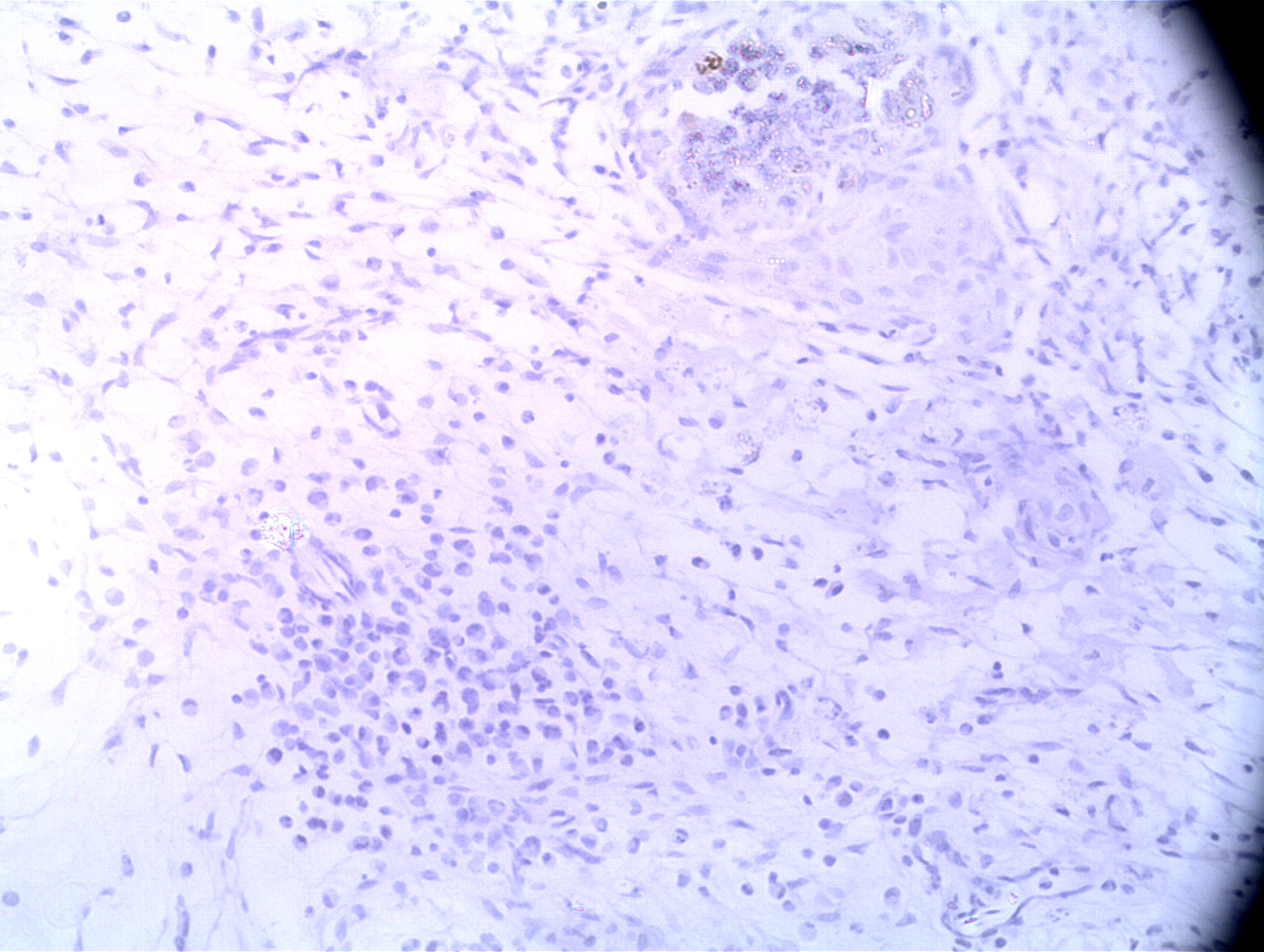

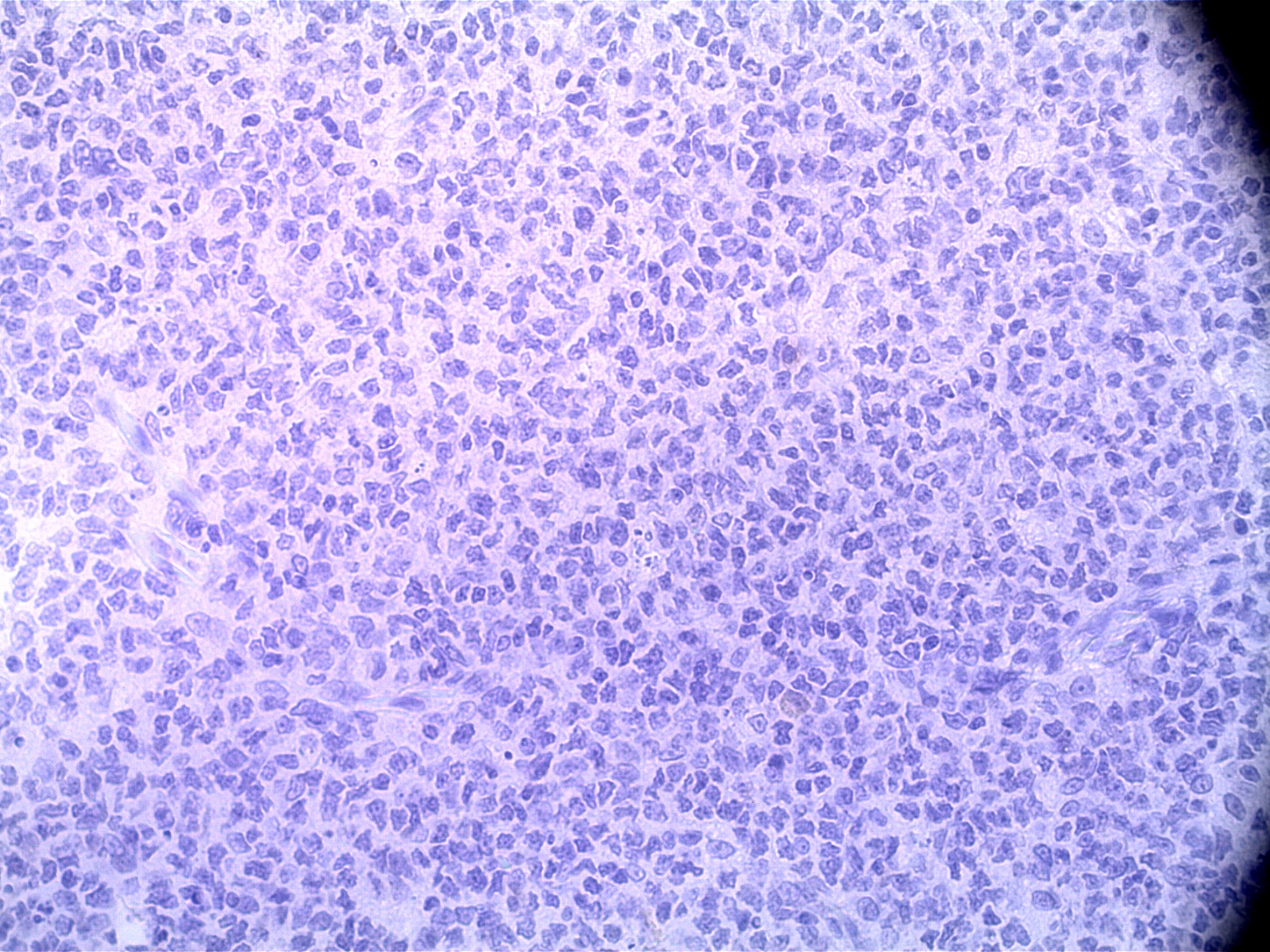

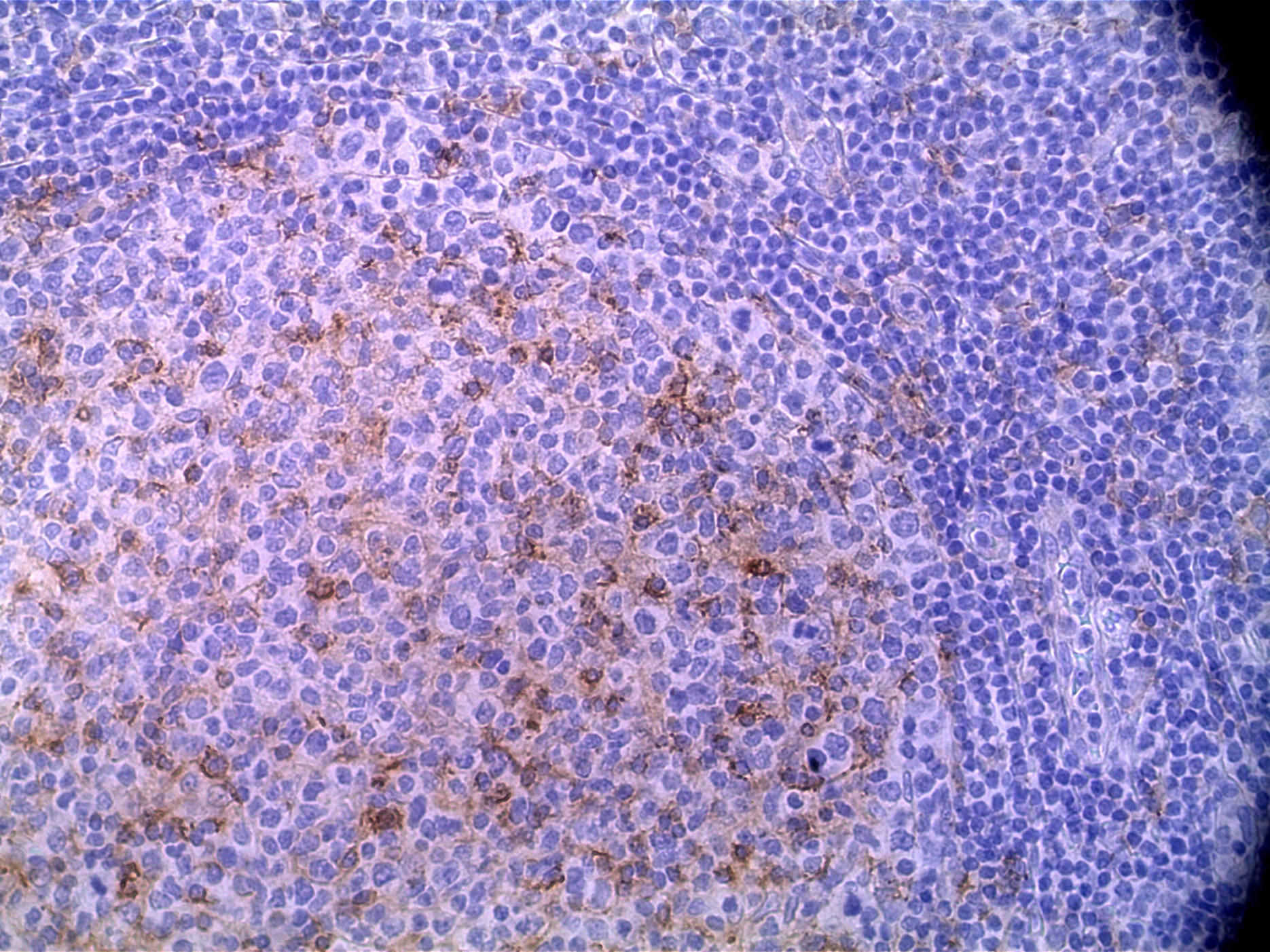

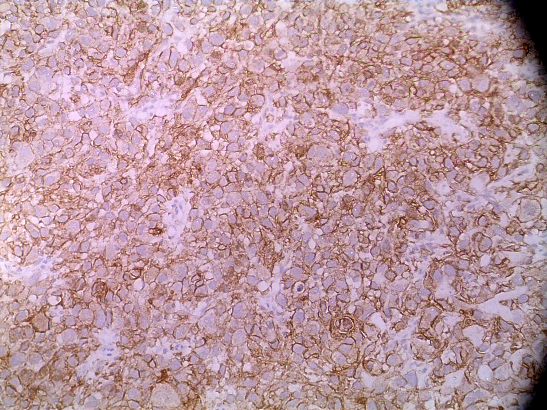

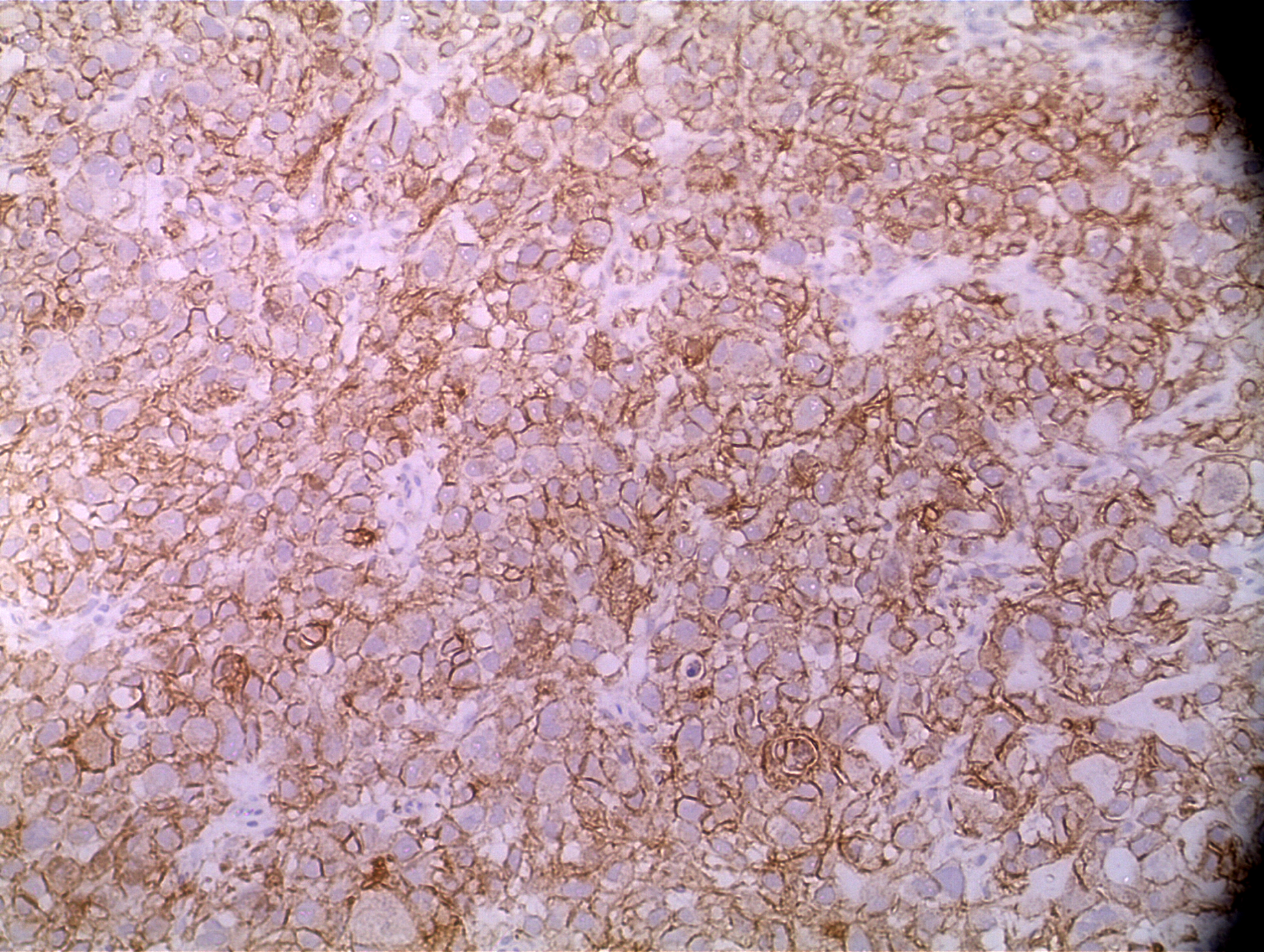


A

RH30 B7H3 KO

Isotype Control

**Additional file 1: Figure S1. Validation of antibody specificity for PD-L1 and B7-H3 staining.**

A) B7H3 staining (D9M2L clone) of wild-type and B7H3 knockout RH30 (rhabdomyosarcoma) tumor xenografts

B) Staining of human tonsil with antibody-matched isotype and PD-L1 antibody (E1L3N clone)

PD-L1 (E1L13N)

B

RH30 wild-type

**Additional file 1: Figure S2. Concordance correlation coefficient between PD-L1 and B7-H3 H-score in tumor cells measured by two independent pathologists**

Dot plots showing the degree of concordance in H-scoring between two pathologists for PD-L1 (left panel) and B7-H3 (right panel).

**Additional file 1: Materials**

Immunohistochemical Staining: PD-L1 (CD274)

Tissue sectioning and IHC staining was performed at the Pathology Research Core (Mayo Clinic, Rochester, MN) using the Leica Bond RX stainer (Leica). FFPE tissues were sectioned at 5 microns and IHC staining was performed on-line. Slides were retrieved for 20 minutes using Epitope Retrieval 2 (EDTA; Leica) and incubated in Protein Block (Dako) for 5 minutes. The PD-L1 (clone E1L3N), rabbit monoclonal antibody from Cell Signaling was diluted to 1:400 in Background Reducing Diluent (Dako) and incubated for 15 minutes.

The detection system used was Polymer Refine Detection System (Leica). This system includes the hydrogen peroxidase block, post primary and polymer reagent, DAB, and Hematoxylin. Immunostaining visualization was achieved by incubating slides 10 minutes in DAB and DAB buffer (1:19 mixture) from the Bond Polymer Refine Detection System. To this point, slides were rinsed between steps with 1X Bond Wash Buffer (Leica). Slides were counterstained for five minutes using Schmidt hematoxylin and molecular biology grade water (1:1 mixture), followed by several rinses in 1X Bond wash buffer and distilled water, this is not the hematoxylin provided with the Refine kit. Once the immunochemistry process was completed, slides were removed from the stainer and rinsed in tap water for five minutes. Slides were dehydrated in increasing concentrations of ethyl alcohol and cleared in 3 changes of xylene prior to permanent coverslipping in xylene-based medium.

Immunohistochemical Staining: B7H3 (CD276)

FFPE tissues were sectioned at 5 microns and tissue sections were deparaffinized in xylene and rehydrated through decreasing gradient of ethyl alcohol. For antigen retrieval, sections were heated in a 10mM citrate buffer (pH6.0) for 20 min at 95°C in a steamer. After cooling to room temperature, endogenous peroxidase activity was quenched with 0.3% of hydrogen peroxidase blocking reagent (Abcam, ab64218) for 10 min at room temperature. After TBST washes, nonspecific antibody binding was blocked by preincubating slides with 3% BSA + 10% normal goat serum at room temperature for 30 min. Slides were then incubated overnight at 4^o^C with anti-B7H3 antibody (1:1000, Cell Signaling; D9M2L). After TBST washes, sections were incubated with biotinylated goat anti-mouse anti-rabbit (Abcam, ab64257) for 15 min at room temperature. After incubating streptavidin peroxidase (Abcam, ab64269) for 15 min at room temperature, the sections were developed with diaminobenzidine (DAB) substrate (Abcam, ab64238). Sections were washed in running tap water and lightly counterstained with hematoxylin, followed by dehydration in increasing concentrations of ethyl alcohol prior to permanent coverslipping (Sigma, 06522).
